# Mouse BMD Quantitative Trait Loci Show Improved Concordance With Human Genome-wide Association Loci When Recalculated on a New, Common Mouse Genetic Map

**DOI:** 10.1002/jbmr.72

**Published:** 2010-02-23

**Authors:** Cheryl L Ackert-Bicknell, David Karasik, Qian Li, Randy V Smith, Yi-Hsiang Hsu, Gary A Churchill, Beverly J Paigen, Shirng-Wern Tsaih

**Affiliations:** 1 The Jackson LaboratoryBar Harbor, ME, USA; 2 Hebrew SeniorLife and Harvard Medical SchoolBoston, MA, USA

**Keywords:** genetic linkage, quantitative trait loci, mouse, human

## Abstract

Bone mineral density (BMD) is a heritable trait, and in mice, over 100 quantitative trait loci (QTLs) have been reported, but candidate genes have been identified for only a small percentage. Persistent errors in the mouse genetic map have negatively affected QTL localization, spurring the development of a new, corrected map. In this study, QTLs for BMD were remapped in 11 archival mouse data sets using this new genetic map. Since these QTLs all were mapped in a comparable way, direct comparisons of QTLs for concordance would be valid. We then compared human genome-wide association study (GWAS) BMD loci with the mouse QTLs. We found that 26 of the 28 human GWAS loci examined were located within the confidence interval of a mouse QTL. Furthermore, 14 of the GWAS loci mapped to within 3 cM of a mouse QTL peak. Lastly, we demonstrated that these newly remapped mouse QTLs can substantiate a candidate gene for a human GWAS locus, for which the peak single-nucleotide polymorphism (SNP) fell in an intergenic region. Specifically, we suggest that *MEF2C* (human chromosome 5, mouse chromosome 13) should be considered a candidate gene for the genetic regulation of BMD. In conclusion, use of the new mouse genetic map has improved the localization of mouse BMD QTLs, and these remapped QTLs show high concordance with human GWAS loci. We believe that this is an opportune time for a renewed effort by the genetics community to identify the causal variants regulating BMD using a synergistic mouse-human approach. © 2010 American Society for Bone and Mineral Research.

## Introduction

Currently, 43.7 million Americans over the age of 50 years are predicted to already have or be at serious risk of developing osteoporosis.(1) Bone mineral density (BMD) is a clinically measurable predictor of future fracture risk, and studies in humans have demonstrated that over 80% of the variance in peak bone mass is due to heritable factors.(2) Human genome-wide association studies (GWAS) have identified several significant loci for BMD, but collectively, the genome-wide significant (GWS) loci identified to date explain only a small portion of the variance in BMD.(3) This suggests that many more loci may be involved in the regulation of BMD.(4) Furthermore, identification of a locus by GWAS does not necessarily equal identification of the causal variant/gene.(5) For other complex phenotypes, such as high-density lipoprotein (HDL) cholesterol, it has been demonstrated that genetic studies in mice are very powerful tools for candidate gene identification in humans.(6) To date, well over 100 quantitative trait loci (QTLs) mapped in mice for BMD are reported in the literature, but few of the underlying genes have been identified.(7)

The QTLs found in single-cross analyses typically have confidence intervals (CIs) extending for 20 to 40 cM, encompassing hundreds of genes,(8) Two powerful techniques have been developed to combine information from multiple QTLs to narrow the list of candidate genes: combined cross-analysis and block haplotyping. Combined cross-analysis uses two or more crosses for which a QTL for the same phenotype has been mapped at the same or similar genetic location. The data from these crosses are merged, and the QTLs are remapped.(8) This technique improves both the power for QTL detection and QTL resolution.(9,10) Block haplotyping is the second method used to narrow QTLs. When a QTL is found, it is safe to assume that the gene responsible for the QTL will not be located where the two parental strains share a haplotype (ie, are genetically identical).(11) Instead, the gene will be located in a haplotype block for which the two strains are dissimilar. When a QTL has been identified in two distinct crosses at the same location, it therefore can be assumed that the gene will be in a region where the two parental strains carrying the high allele share a haplotype, where the two parental strains carrying the low allele share a haplotype, and where the high-allele and low-allele haplotypes are different. This block haplotyping approach can be used to reduce the size of the QTL region by up to 90%.(9,11,12)

Both these techniques rely on knowing what other QTLs have been mapped for a given phenotype and exactly where those QTLs are. Unfortunately, obtaining complete and accurate information about bone QTLs mapped in mice is not as straightforward as a simple literature search. The type and level of information published about the QTLs have improved along with the mapping techniques. Often, suggestive QTLs (ie, QTLs for which the significance level was above background but did not reach a *p* < .05 significance) are not reported for fear of “cluttering the literature.” Suggestive QTLs, nonetheless, can be used for both block haplotyping and combined cross-analysis to increase the power of detection and narrow the QTL. Pertinent information for a given QTL may never have been reported. Specifically, CIs or allele effects, both of which are required for block haplotyping, are often missing. Also, the marker closest to the peak may have been reported instead of the location of the actual QTL peak location. Depending on the density of the markers used in actually genotyping the cross, the marker closest to the peak and the actual peak of the QTL could be several cM apart. The algorithm used for QTL mapping often varies widely among studies, and different covariates such as body weight may or may not have been incorporated into the model, making absolute comparison of a pair of QTLs difficult. The last factor hindering the comparison of QTLs found in different studies is the fact that there are problems with the standard mouse genetic map.

The current standard genetic map for the mouse is curated and maintained by the Mouse Genome Informatics (MGI) Group at the Jackson Laboratory (http://www.informatics.jax.org/).(13) This map is based largely on information obtained from two small mouse crosses.(14,15) The traditional genetic map is updated on an annual basis and has improved over time,(16) but it still retains historical errors related to marker order and chromosome assignment.(17) Mapping QTLs requires accurate genetic map information for both the relative order of markers and the distances between them. Recently, Shifman and colleagues published a new genetic map based on a large heterogeneous stock population.(18) A total of 7080 standard simple-sequence-length polymorphism (SSLP) markers were integrated to this single-nucleotide polymorphism (SNP)–based map, generating a corrected mouse genetic map.(17) This new map resolves inconsistencies between the physical and genetic maps and provides highly accurate genetic distances. A recent mapping study, in which the new and traditional genetic maps were compared, suggests that up to 20% of published QTLs may have been mislocalized owing to marker order and positioning errors in the old genetic map.(17) In this study we have collected the raw data from the majority of the original mouse crosses for bone phenotypes. The QTLs for the BMD phenotypes measured in each cross were remapped using the new mouse genetic map. Lastly, the mouse QTL peak locations were compared with human BMD GWAS loci.

## Materials and Methods

### Identification of mapping-cross data sets

First, a literature search to identify published reports of mouse BMD QTLs was done using the following key words: *BMD, mouse, QTL, bone mineral density, mapping*, and/or *loci*. Second, the Mouse Genome Informatics Database (http://www.informatics.jax.org/) was searched for bone-related QTLs using the “Genes and Markers Query” form. Specifically, this database was searched using the keyword *bone* in the “Gene/Marker Symbol/Name” field, *QTL* in the “Type” field, and *Any* in the “Chromosome” field; the “No Limit” box was checked under the heading of “Maximum Returned.” QTLs for non-BMD phenotypes, such as “bone marrow graft rejection” and “bone area,” were struck from the returned list. The cross in which each BMD QTL was mapped was identified and compared with the list of crosses identified by conventional literature search. In short, the result was a list of mouse mapping crosses in which BMD QTLs were mapped regardless of the method used to measure density.

### Main-effects QTL mapping

Map positions for the markers for all data sets were updated to the new mouse genetic map using a mouse map converter tool found at http://cdg.jax.org. All QTL analyses were done using the R/qtl software package(19) (R Version 2.6.2, qtl Library Version 1.09-43, http://www.rqtl.org/). Phenotypic outliers were removed prior to QTL analyses. In all cases, the phenotype trait data were transformed using the van der Waerden normal score method to correct for any skewing of the data.(20) Since we did not generate the original data, we performed rigorous quality-control analysis of the genotyping data prior to QTL mapping. First, errors in genotyping were identified by comparing the “User imputed marker positions” (ie, marker positions in cM from the new mouse genetic map) with the cross-calculated genetic position, as calculated in a cross-specific manner, using R/qtl. When the cross-calculated genetic positions were in large disagreement with marker positions input by the user, possible errors in marker ordering were examined. Second, the recombination fraction for all possible pairs of markers for a given cross were calculated using the *est.rf* function in R/qtl. The recombination fractions then were plotted using the *plot.rf* function in R/qtl. Markers exhibiting low recombination fraction/high LOD with nonadjacent markers (ie, markers at the opposite end of the same chromosome or with a marker on a completely different chromosome) were considered to be in error. When marker errors could not be resolved, the marker was deleted. A single-locus mainscan for QTL was performed, and LOD scores were calculated at 2-cM intervals across the genome using the “imp,” or imputation, method in R/qtl for all data sets except the B6xC3H data set from Farber and colleagues.(21) The data set from Farber and colleagues included genotyping for 1486 markers, with no missing genotypes. Because of this high density in genotyping data, marker regression was run for the main QTL scan for this one data set. The LOD thresholds for significant and suggestive QTLs were determined in a cross-specific manner based on 1000 permutations of the data.(22) A QTL was considered to be suggestive if the LOD score exceeded the *p* < .63 threshold and significant if it exceeded the *p* < .05 threshold. These thresholds were chosen because they are the widely accepted cutoffs for suggestive and significant QTLs.(23) The goal of this analysis was to identify main-effect QTLs; therefore, no pairwise/interactive genome scans were run.

For 5 of the 12 crosses, data were available for both male and female mice (Table 1). To account for the average differences between the males and females, we carried out scans using sex as an additive covariate. To identify sex-dependent QTL effects, we carried out additional scans using sex as an interactive covariate and computed the differences in LOD scores between these two scans (ie, the ΔLOD).(24) The interactive scan model identified the most likely position of the sex-specific QTLs. Calculating the ΔLOD score at the peak position is the secondary test for the QTL-by-sex interaction.(24) This secondary test is carried out with no adjustment for multiple testing, and the threshold, based on the usual chi-square distribution of the likelihood ratio, is 2.0 on the LOD scale. The sex specificity of these QTLs then was confirmed using the *addqtl* function in R/qtl by including “Q_*i*_:sex” in the main effected model, where *i* was the location of the putative sex-specific QTL. The cross generated by Farber and colleagues was bred in a reciprocal fashion (ie, B6 × C3H F_1_ × C3H × B6 F_1_), and therefore, cross-direction also was considered in the main effects model. Similar to the sex covariate, the strain of paternal grandmother (pgm) for each mouse was considered as both an additive and interactive covariate. The ΔLOD was calculated to identify potential “pgm-specific QTLs.” The covariate of “sex:pgm” also was included in the model to look for QTLs that were contingent on both cross-direction and gender. The pgm specificity of these QTLs was confirmed using the *addqtl* function, including “Q_*i*_:pgm” in the model, as was described for sex-specific QTLs.

**Table 1:** Description of the Cross Data Sets

Cross (reference)	Number of mice^a^	Phenotype(s) examined	Age (weeks)	Notes^b^
B6 × 129(35)	291 (F)	Whole-body aBMD	20	Fed HF for last 14 weeks
		Vertebral aBMD		
B6 × C3H(34,54)	998 (F)	Femoral vBMD	16	
		Vertebral vBMD		
B6 × C3H(29)	145 (F)	Femoral aBMD	35	Fed HF for last 14 weeks
	164 (M)			
B6 × CASA(52)	184 (F)	Whole-body aBMD	11	
	185 (M)			
B6 × CAST(36)	711 (F)	Femoral vBMD	16	Selectively genotyped
B6 × DBA(55–57)	595 (F)	Whole-body aBMD	16	Data available for 2 ages, only used younger mice in this analysis
	391 (M)	Femoral aBMD		
B6 × DBA(30)	110 (F)	Femoral aBMD	68	Fed HF for last 16 weeks
		Femoral vBMD		
		Midshaft vBMD		
MRL × CAST(39)	170 (F)	Whole-body aBMD	7	
	157 (M)	Midshaft vBMD		
MRL × SJL(32,33)	621 (F)	Whole-body aBMD	7	
		Midshaft vBMD		
NZB × RF(31)	661 (F)	Femoral vBMD	10	
		Femoral cortical vBMD		
		Midshaft vBMD		
		Midshaft cortical vBMD		
NZB × SM(58)	143 (F)	Whole-body aBMD	24	Fed HF for last 16 weeks
	124 (M)	Vertebral aBMD		

a F = female; M = male.

b HF = high-fat diet.

### Comparison with human BMD genetic loci

Human BMD genome-wide significant loci from GWAS studies were identified from the literature.(3,25–27) For each GWS locus, dbSNP (Build 130) was used to determine the genomic coordinates for the most significant SNP and 500 bp surrounding the SNP. These human genomic coordinates were lifted over to the mouse genome (Build 37) using the “Batch Coordinate Conversion” tool found at http://genome.ucsc.edu/index.html. If the Batch Coordinate Conversion lift-over was unsuccessful, the region surrounding the SNP was examined in the human genome browser window. The interval examined was expanded 1.5-fold until a conserved mouse/human sequence was identified on the “Mouse (July 2007/mm^9^) Alignment Net” track. The coordinates in the mouse genome for this conserved track were extracted. All mouse genomic coordinates then were converted to the cM position using the “Mouse Map Conversion Tool” found at http://cgd.jax.org/.

We wished to confirm that lifting over the genomic coordinates of the most significant SNPs from human to mouse meant moving over to the same gene “neighborhood” in both human and mouse. Therefore, we identified the mouse gene that was homologous to the human gene closest to the GWS peak. We then identified the chromosome, starting Mb and ending Mb, in the mouse for that gene. In all cases, we found that the coordinates for the peak SNP, as lifted over into mouse, were located in close proximity to the identified homologous mouse gene. This indicated that we were correctly moving the human GWAS peaks over to a similar gene neighborhood in the mouse.

### Block haplotyping

Block haplotyping was done using the strain-comparison tool located at http://cgd.jax.org/. Specifically, the “Imputed Diversity Array, Build 37” SNP set was used. The following comparison expression was built:


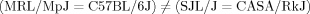
(1)

All blocks consisting of at least one SNP extending for at least 1 bp were extracted. The results for all of chromosome 13 were downloaded, and any blocks outside our region of interest were deleted. The start and end positions for all mouse *Refseq* genes found in our region of interest were downloaded from the UCSC genome browser (http://genome.ucsc.edu). A gene was considered a candidate if a haplotype block was found anywhere between the genomic start and end positions for that gene. Since CASA/RkJ is a wild-derived strain, we were concerned that we were inappropriately removing genes from the candidate list by forcing SJL/J to equal CASA/RkJ in the haplotype block definition. We then repeated this exercise without the SJL/J equal to the CASA/RkJ term.

## Results

### Eligible data sets for reanalysis

Collectively, 15 mouse-mapping crosses in which BMD QTLs had been mapped were identified. The corresponding author and senior author for the first paper describing the data set were contacted and asked if they were willing to provide complete genotype and phenotype data for their data set for a mouse BMD QTL remapping project. Authors were asked separately if they were willing to deposit their data set into a public QTL data repository, such as the Center for Genome Dynamic's QTL archive (http://cgd.jax.org/nav/qtlarchive1.shtml). In total, 13 data sets of the 15 requested were received. No response was received from one set of authors, and one data set was not available for analysis. We decided not to include two data sets that were received for this project because they were too small and/or incomplete to do accurate mapping. The first of these two crosses(28) did not have genotype information available for every chromosome and had very low marker density (one to three markers per chromosome) for other chromosomes. The second of these(28) had only had 51 mice with genotype information available, making this data set underpowered for QTL detection in the algorithm we used. In sum, 11 data sets were used for this mouse BMD QTL recalculation project (Table 1).

### Overview of QTLs mapped

**Fig. 1:**
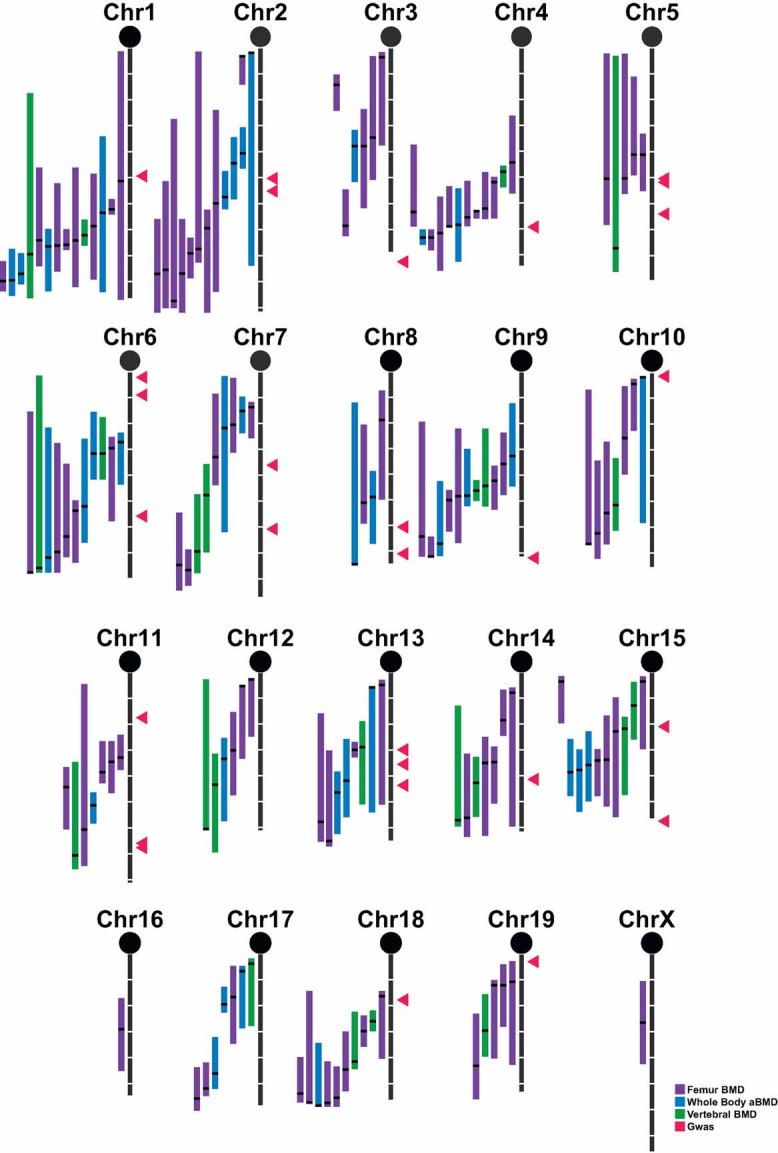
Mouse BMD QTLs compared with human GWAS loci. BMD QTLs for femoral (*purple*), whole-body (*blue*), and vertebral (*green*) BMD are presented to scale on the left side of each chromosome. Each white space along the chromosomal axis represents 10 cM. Each QTL is presented such that the vertical bar representing the QTL starts at the upper limit of the 95% confidence interval (CI) and extends to the lower end of the CI. The peak for the QTL is denoted by a black bar within the CI span. The human GWAS loci, as lifted over to the mouse genome coordinates, are represented as red triangles to the right of each chromosome.

In total, 155 primary QTLs were identified in the 11 data sets used for analysis, as is presented in Table 2: 86 QTLs for femoral BMD, 34 for vertebral BMD, and 35 for whole-body areal BMD (aBMD). At least one QTL for BMD was identified for each chromosome, with the exception of the Y chromosome (Fig. 1). We identified six QTLs that were specific to males, six QTLs that were specific to females, and 47 QTLs that were not sex specific. An additional 96 QTLs were mapped in crosses for which only data from female mice were available, and thus no determination of sex specificity could be made. One QTL, on chromosome 7, mapped in the B6 × C3H data set from Farber and colleagues,(29) was found to be cross-direction-specific, indicating a potential parent-of-origin effect.

**Table 2:** Main Scan QTL Peaks Identified

Chr	Peak (cM)	LOD score	Confidence interval (cM)	Peak marker	Phenotype^a^	High allele	Cross (reference)	Gender specific^b^
1	51.66	2.13	1.66–97.30	*D1Mit191*	Femur aBMD	B6	B6 × DB(56,57)	F & M
1	62.50	27.50	58.50–64.50	*D1Mit14*	Femur vBMD	C3H	B6 × C3H(34)	F (M ?)
1	63.84	3.23	34.46–94.35	*D1Mit346*	Whole-body aBMD	MRL	MRL × SJL(32,33)	F (M ?)
1	69.01	3.41	48.81–89.51	*rs3701299*	Femur aBMD	C3H	B6 × C3H(29)	F & M
1	72.50	14.04	66.50–76.50	*D1Mit14*	Vertebral vBMD	C3H	B6 × C3H(34)	F (M ?)
1	74.46	2.32	46.46–92.46	*D1Mit111*	Femoral midcort vBMD	NZB	NZB × RF(31)	F (M ?)
1	74.46	3.51	44.46–84.46	*D1Mit111*	Femoral mid vBMD	NZB	NZB × RF(31)	F (M ?)
1	76.12	10.96	70.12–78.12	*D1Mit15*	Femur vBMD	CAST	B6 × CAST(36)	F (M ?)
1	76.46	2.67	52.46–86.46	*D1Mit111*	Femur vBMD	NZB	NZB × RF(31)	F (M ?)
1	76.73	4.06	70.08–94.08	*D1Mit540.1*	Whole-body aBMD	CASA	B6 × CASA(52)	F & M
1	80.08	3.13	18.08–97.04	*D1Mit115*	Vertebral aBMD	129	B6 × 129(59)	F (M ?)
1	87.66	4.49	79.66–91.66	*D1Mit291*	Whole-body aBMD	DBA	B6 × DBA(56,57)	F & M
1	90.08	3.96	78.08–96.08	*D1Mit406*	Whole-body aBMD	129	B6 × 129(35)	F (M ?)
1	90.46	5.87	82.46–94.35	*D1Mit291*	Femoral mid vBMD	MRL	MRL × SJL(32,33)	F (M ?)
2	2.23	2.62	2.23–84.23	*D2Mit1*	Whole-body aBMD	MRL	MRL × CAST(39)	F & M
2	3.62	3.64	3.62–14.54	*rs3676722*	Femur aBMD	B6	B6 × C3H(29)	M
2	40.90	4.12	30.90–46.90	*D2Mit205*	Whole-body aBMD	B6	B6 × CASA(52)	F & M
2	44.70	8.28	34.70–58.70	*D2Mit91*	Whole-body aBMD	DBA	B6 × DBA(56,57)	F
2	57.81	4.32	47.81–62.49	*D2Mit62*	Whole-body aBMD	MRL	MRL × SJL(32,33)	F (M ?)
2	60.23	1.75	24.23–94.23	*D2Mit46*	Femur vBMD	B6	B6 × CAST(36)	F (M ?)
2	69.80	2.72	62.70–102.29	*D2Mit166*	Femur aBMD	DBA	B6 × DBA(56,57)	F
2	77.81	2.15	1.81–82.95	*D2Mit285*	Femoral mid vBMD	SJL	MRL × SJL(32,33)	F (M ?)
2	79.42	4.04	73.42–88.99	*D2Mit48*	Femur vBMD	C3H	B6 × C3H(34)	F (M ?)
2	85.81	2.29	51.81–100.49	*D2Mit411*	Femoral mid vBMD	RF	NZB × RF(31)	F (M ?)
2	87.22	2.57	66.23–102.23	*D2Mit413*	Femur aBMD	B6	B6 × DBA(30)	F (M ?)
2	87.22	2.57	66.23–102.23	*D2Mit413*	Femur aBMD	B6	B6 × DBA(30)	F (M ?)
2	97.81	2.63	27.81–100.49	*D2Mit148*	Femoral midcort vBMD	RF	NZB × RF(31)	F (M ?)
3	3.96	2.83	1.96–37.96	*D3Mit23*	Femur vBMD	CAST	B6 × CAST(36)	F (M ?)
3	14.82	4.95	10.82–24.82	*D3Mit203*	Femoral mid vBMD	RF	NZB × RF(31)	F (M ?)
3	34.85	2.55	3.55–51.21	*rs3714671*	Femur aBMD	C3H	B6 × C3H(29)	F & M
3	38.19	2.74	24.01–62.01	*D3Mit40*	Femur aBMD	B6	B6 × DBA(56,57)	F & M
3	38.19	3.93	32.01–52.01	*D3Mit40*	Whole-body aBMD	B6	B6 × DBA(56,57)	F & M
3	68.85	4.00	54.85–72.85	*D3Mit127*	Femur vBMD	DBA	B6 × DBA(30)	F (M ?)
4	44.43	2.96	26.43–56.43	*D4Mit9*	Femoral mid vBMD	MRL	MRL × CAST(39)	F & M
4	48.04	15.07	45.76–54.04	*D4Mit187*	Vertebral vBMD	C3H	B6 × C3H(34)	F (M ?)
4	52.04	22.94	50.04–66.04	*D4Mit124*	Femur vBMD	C3H	B6 × C3H(34)	F (M ?)
4	62.14	2.82	48.14–66.26	*D4Mit204*	Femoral mid vBMD	SJL	MRL × SJL(32,33)	F (M ?)
4	63.26	12.22	63.26–66.01	*rs3023007*	Femur aBMD	C3H	B6 × C3H(29)	F & M
4	63.60	2.64	37.60–69.05	*D4Mit251*	Femur vBMD	RF	NZB × RF(31)	F (M ?)
4	65.60	4.28	51.60–69.05	*D4Mit251*	Femoral cort vBMD	RF	NZB × RF(31)	F (M ?)
4	68.43	3.04	54.43–82.64	*D4Mit308*	Whole-body aBMD	129	B6 × 129(35)	F (M ?)
4	69.05	4.32	53.60–69.05	*D4Mit251*	Femoral midcort vBMD	RF	NZB × RF(31)	F (M ?)
4	71.60	2.45	57.60–86.17	*D4Mit68*	Femur vBMD	B6	B6 × CAST(36)	F (M ?)
4	73.41	4.94	70.22–78.22	*D4Mit48*	Femur aBMD	DBA	B6 × DBA(56,57)	F & M
4	73.41	10.94	70.22–76.22	*D4Mit48*	Whole-body aBMD	DBA	B6 × DBA(56,57)	F & M
5	41.43	4.26	11.43–49.43	*D5Mit10*	Femoral mid vBMD	MRL	MRL × CAST(39)	F
5	41.43	5.06	33.43–55.43	*D5Mit112*	Femur vBMD	B6	B6 × CAST(36)	F (M ?)
5	50.62	3.47	2.62–56.62	*D5Mit10*	Femoral midcort vBMD	RF	NZB × RF(31)	F (M ?)
5	50.64	2.62	2.621–68.62	*D5Mit10*	Femoral mid vBMD	RF	NZB × RF(31)	F (M ?)
5	77.43	2.44	3.43–86.57	*D5Mit284*	Vertebral aBMD	SM	NZB × SM(58)	F & M
6	27.38	3.88	23.81–43.81	*D6Mit384*	Whole-body aBMD	B6	B6 × CASA(52)	F & M
6	29.81	4.03	25.40–57.81	*D6Mit124*	Femur vBMD	B6	B6 × C3H(34)	F (M ?)
6	31.81	3.61	17.81–41.81	*D6Mit209*	Vertebral aBMD	129	B6 × 129(35)	F (M ?)
6	31.81	4.18	15.81–41.81	*D6Mit209*	Whole-body aBMD	129	B6 × 129(35)	F (M ?)
6	52.15	4.41	26.15–66.15	*D6MIT256*	Whole-body aBMD	MRL	MRL × SJL(32,33)	F (M ?)
6	53.81	3.95	49.81–73.81	*D6Mit55*	Femur aBMD	B6	B6 × DBA(56,57)	M
6	63.74	4.21	35.74–71.74	*D6Mit25*	Femur aBMD	B6	B6 × DBA(30)	F (M ?)
6	69.74	2.57	27.74–77.70	*D6Mit198*	Femur vBMD	DBA	B6 × DBA(30)	F (M ?)
6	71.81	2.25	21.81–77.70	*D6Mit15*	Whole-body aBMD	DBA	B6 × DBA(56,57)	F & M
6	75.81	1.97	1.81–77.70	*D6Mit15*	Vertebral vBMD	B6	B6 × C3H(34)	F (M ?)
6	77.64	2.08	15.81–77.64	*D6Mit14*	Femoral mid vBMD	NZB	NZB × RF(31)	F (M ?)
7	13.94	7.53	11.94–25.94	*D7Mit114*	Femur aBMD	DBA	B6 × DBA(56,57)	F & M
7	15.42	3.44	9.94–23.94	*D7Mit114*	Whole-body aBMD	DBA	B6 × DBA(56,57)	F & M
7	16.94	2.93	8.83–43.97	*rs4226499*	Femur aBMD	C3H	B6 × C3H(29)	F & M
7	20.71	2.21	2.71–31.44	*D7Mit80*	Femoral mid vBMD	B6	B6 × DBA(30)	F (M ?)
7	22.02	2.84	2.02–62.02	*D7Mit310*	Whole-body aBMD	CASA	B6 × CASA(52)	F & M
7^c^	33.16	4.16	8.83–43.97	*rs3688333*	Femur aBMD	C3H	B6 × C3H(29)	F & M
7	47.86	2.90	35.86–69.86	*D7Mit300*	Vertebral aBMD	B6	B6 × 129(35)	F (M ?)
7	69.46	4.84	47.46–77.87	*D7Mit238*	Vertebral vBMD	B6	B6 × C3H(34)	F (M ?)
7	74.71	4.52	54.71–84.71	*D7Mit358*	Femoral mid vBMD	NZB	NZB × RF(31)	F (M ?)
7	76.71	5.16	68.71–82.71	*D7Mit358*	Femur vBMD	NZB	NZB × RF(31)	F (M ?)
8	18.89	1.98	7.59–49.59	*D8Mit4*	Femur vBMD	C3H	B6 × C3H(34)	F (M ?)
8	48.52	2.79	38.52–66.52	*D8Mit211*	Whole-body aBMD	B6	B6 × CASA(52)	F & M
8	50.75	3.81	20.75–58.75	*D8Mit113*	Femur aBMD	DBA	B6 × DBA(56,57)	F & M
8	74.46	2.27	12.14–74.46	*D8Mit42*	Whole-body aBMD	SM	NZB × SM(58)	F & M
9	32.76	2.68	12.46–44.46	*D9Mit207*	Whole-body aBMD	MRL	MRL × SJL(32,33)	F (M ?)
9	35.80	2.24	23.80–47.80	*D9Mit129*	Femoral mid vBMD	CAST	MRL × CAST(39)	F & M
9	42.32	3.82	36.46–53.48	*D9MIT263*	Femoral mid vBMD	MRL	MRL × SJL(32,33)	F (M ?)
9	44.24	4.83	22.24–52.24	*D9Mit196*	Vertebral vBMD	B6	B6 × C3H(34)	F (M ?)
9	46.08	12.54	42.08–50.08	*D9Mit196*	Vertebral aBMD	NZB	NZB × SM(58)	F
9	48.08	9.90	30.08–52.08	*D9Mit196*	Whole-body aBMD	NZB	NZB × SM(58)	F
9	48.24	2.35	22.24–66.24	*D9Mit196*	Femur vBMD	B6	B6 × C3H(34)	F (M ?)
9	49.80	5.49	45.80–61.80	*D9Mit10*	Femur aBMD	B6	B6 × DBA(56,57)	F & M
9	63.8	2.34	19.80–71.49	*D9Mit18*	Femoral mid vBMD	NZB	NZB × RF(31)	F (M ?)
9	66.48	2.95	42.48–71.32	*D9Mit311*	Whole-body aBMD	B6	B6 × CASA(52)	M
9	71.49	6.50	63.80–71.49	*D9Mit18*	Femur vBMD	NZB	NZB × RF(31)	F (M ?)
10	2.53	2.58	2.53–58.53	*D10Mit75*	Whole-body aBMD	B6	B6 × DBA(56,57)	F & M
10	5.03	3.12	3.03–23.03	*D10Mit28*	Femur vBMD	CAST	B6 × CAST(36)	F (M ?)
10	25.91	3.10	5.91–39.91	*D10Mit31*	Femoral mid vBMD	CAST	MRL × CAST(39)	F & M
10	51.66	2.50	33.66–61.58	*D10Mit95*	Vertebral vBMD	C3H	B6 × C3H(34)	F (M ?)
10	54.72	2.95	27.31–66.75	*rs3672179*	Femur aBMD	B6	B6 × C3H(29)	F & M
10	62.53	2.39	34.53–72.31	*D10Mit162*	Femur aBMD	B6	B6 × DBA(56,57)	M
10	66.75	2.30	7.27–66.75	*D10Mit14*	Femoral mid vBMD	NZB	NZB × RF(31)	F (M ?)
11	33.04	6.20	24.20–37.81	*rs4228731*	Femur aBMD	C3H	B6 × C3H(29)	M
11	34.70	6.81	26.70–46.70	*D11Mit242*	Femur vBMD	C3H	B6 × C3H(34)	F (M ?)
11	38.70	7.19	26.70–42.88	*D11Mit30*	Femur vBMD	RF	NZB × RF(31)	F (M ?)
11	44.7	8.49	36.70–60.70	*D11Mit30*	Femoral mid vBMD	RF	NZB × RF(31)	F (M ?)
11	51.34	8.23	46.44–58.44	*D11Mit355*	Whole-body aBMD	B6	B6 × DBA(56,57)	F & M
11	60.70	2.82	4.70–74.70	*D11Mit126*	Femoral midcort vBMD	RF	NZB × RF(31)	F (M ?)
11	70.70	2.92	34.70–75.93	*D11Mit301*	Vertebral vBMD	C3H	B6 × C3H(34)	F (M ?)
12	2.94	4.60	2.94–24.94	*D12Mit215*	Femur vBMD	B6	B6 × C3H(34)	F (M ?)
12	5.52	2.91	5.52–33.52	*D12Mit182*	Femoral mid vBMD	SJL	MRL × SJL(32,33)	F (M ?)
12	30.20	3.52	15.52–47.52	*D12Mit201*	Femur vBMD	NZB	NZB × RF(31)	F (M ?)
12	33.52	6.32	25.52–57.52	*D12Mit60*	Whole-body aBMD	NZB	NZB × SM(58)	F & M
12	43.52	5.24	31.52–69.52	*D12Mit60*	Vertebral aBMD	NZB	NZB × SM(58)	F & M
12	60.56	2.60	2.94–60.56	*D12Mit79*	Vertebral vBMD	C3H	B6 × C3H(34)	F (M ?)
13	5.08	3.33	3.08–51.11	*D13Mit205*	Femur vBMD	CAST	B6 × CAST(36)	F (M ?)
13	6.05	2.18	6.05–54.05	*D13Mit303*	Whole-body aBMD	DBA	B6 × DBA(56,57)	F & M
13	28.99	2.81	18.99–50.99	*D13Mit13*	Vertebral vBMD	B6	B6 × C3H(34)	F (M ?)
13	30.06	11.19	26.99–32.99	*D13Mit13*	Femur vBMD	C3H	B6 × C3H(34)	F (M ?)
13	41.92	4.08	25.92–55.92	*D13Mit202*	Whole-body aBMD	MRL	MRL × SJL(32,33)	F (M ?)
13	46.45	5.24	38.45–62.45	*D13Mit191*	Whole-body aBMD	B6	B6 × CASA(52)	F & M
13	57.92	2.24	15.92–65.43	*D13Mit204*	Femoral mid vBMD	NZB	NZB × RF(31)	F (M ?)
13	65.17	2.57	30.31–67.21	*rs3656262*	Femur aBMD	C3H	B6 × C3H(29)	F & M
14	8.03	2.55	6.03–59.53	*D14Mit132*	Femoral mid vBMD	MRL	MRL × SJL(32,33)	F (M ?)
14	18.67	3.04	6.86–24.60	*rs3709612*	Femur aBMD	C3H	B6 × C3H(29)	F & M
14	34.73	4.66	28.73–50.73	*D14Mit160*	Femur vBMD	C3H	B6 × C3H(34)	F (M ?)
14	35.08	2.50	25.08–63.08	*D14Mit142*	Femur aBMD	DBA	B6 × DBA(56,57)	F & M
14	42.73	4.58	32.73–55.83	*D14Mit160*	Vertebral vBMD	C3H	B6 × C3H(34)	F (M ?)
14	56.16	3.66	31.53–63.53	*D14Mit170*	Femur vBMD	CAST	B6 × CAST(36)	F (M ?)
14	57.08	2.05	13.08–59.53	*D14MIT75*	Vertebral aBMD	129	B6 × 129(35)	F (M ?)
15	3.80	3.32	1.80–29.80	*D15Mit13*	Femur aBMD	DBA	B6 × DBA(30)	F (M ?)
15	3.80	2.96	1.80–19.80	*D15Mit13*	Femur aBMD	DBA	B6 × DBA(30)	F (M ?)
15	13.02	2.65	3.96–26.07	*D15Mit111*	Vertebral vBMD	B6	B6 × C3H(34)	F (M ?)
15	21.96	2.24	17.41–47.41	*D15Mit26*	Vertebral aBMD	NZB	NZB × SM(58)	F & M
15	22.92	2.31	9.84–55.99	*D15MIT115*	Femoral mid vBMD	MRL	MRL × CAST(39)	F & M
15	33.84	3.20	16.82–51.84	*D15Mit63*	Femur aBMD	B6	B6 × DBA(56,57)	F & M
15	34.16	4.54	29.80–47.80	*D15Mit29*	Femur vBMD	B6	B6 × CAST(36)	F (M ?)
15	35.84	3.34	22.92–49.84	*D15MIT159*	Whole-body aBMD	MRL	MRL × CAST(39)	F & M
15	37.85	8.28	29.84–53.94	*D15Mit2*	Whole-body aBMD	B6	B6 × CASA(52)	F & M
15	38.67	3.88	25.84–47.84	*D15Mit189*	Whole-body aBMD	B6	B6 × DBA(56,57)	M
16	29.33	4.04	17.33–45.33	*D16Mit12*	Femur vBMD	C3H	B6 × C3H(34)	F (M ?)
17	4.11	2.46	2.11–28.11	*D17Mit164*	Vertebral vBMD	B6	B6 × C3H(34)	F (M ?)
17	6.92	2.54	4.92–28.93	*D17Mit143*	Whole-body aBMD	B6	B6 × DBA(56,57)	F & M
17	16.93	4.16	4.92–34.92	*D17Mit175*	Femoral mid vBMD	MRL	MRL × SJL(32,33)	F (M ?)
17	19.66	5.00	12.93–22.93	*D17Mit176*	Whole-body aBMD	MRL	MRL × SJL(32,33)	F (M ?)
17	46.29	3.38	32.29–52.25	*D17Mit39*	Whole-body aBMD	MRL	MRL × CAST(39)	F & M
17	52.11	3.12	42.11–55.02	*D17Mit155*	Femur vBMD	C3H	B6 × C3H(34)	F (M ?)
17	55.93	2.27	43.93–60.67	*D17Mit123*	Femoral midcort vBMD	NZB	NZB × RF(31)	F (M ?)
18	15.02	3.64	13.02–39.02	*D18Mit34*	Femoral mid vBMD	MRL	MRL × SJL(32,33)	F (M ?)
18	24.64	8.11	20.46–28.46	*D18Mit36*	Vertebral vBMD	C3H	B6 × C3H(34)	F (M ?)
18	28.46	17.19	22.46–34.46	*D18Mit36*	Femur vBMD	C3H	B6 × C3H(34)	F (M ?)
18	40.14	2.83	21.02–43.02	*D18MIT50*	Vertebral aBMD	129	B6 × 129(35)	F (M ?)
18	43.21	3.88	28.09–51.07	*rs3669949*	Femur aBMD	C3H	B6 × C3H(29)	F & M
18	54.04	4.88	40.04–57.53	*D18Mit4*	Femoral midcort vBMD	RF	NZB × RF(31)	F (M ?)
18	54.04	5.49	42.04–57.53	*D18Mit4*	Femoral midcort vBMD	RF	NZB × RF(31)	F (M ?)
18	56.04	2.92	40.04–57.53	*D18Mit4*	Femur vBMD	RF	NZB × RF(31)	F (M ?)
18	57.02	3.32	33.02–57.53	*D18MIT43*	Whole-body aBMD	129	B6 × 129(35)	F (M ?)
18	57.53	1.75	14.53–57.53	*D18Mit6*	Femur vBMD	CAST	B6 × CAST(36)	F (M ?)
19	11.04	1.58	3.04–43.04	*D19Mit32*	Femur vBMD	CAST	B6 × CAST(36)	F (M ?)
19	12.34	3.24	4.34–28.34	*D19Mit16*	Femur aBMD	DBA	B6 × DBA(56,57)	F
19	12.40	2.08	10.40–40.53	*D19Mit61*	Femur vBMD	C3H	B6 × C3H(34)	F (M ?)
19	29.82	2.34	15.82–39.82	*D19Mit11*	Vertebral aBMD	SM	NZB × SM(58)	F & M
19	43.33	2.46	23.33–56.28	*D19Mit53*	Femoral mid vBMD	SJL	MRL × SJL(32,33)	F (M ?)
X	26.73	4.78	10.73–42.73	*DXMit144*	Femur aBMD	B6	B6 × DBA(56,57)	F & M

a Midshaft (mid), cortical (cort).

b Female (F), Male (M); no data available for males in that cross (?).

c Cross-direction-specific, only seen in C3H × B6 cross direction.

For both the B6 × DBA cross generated by Drake and colleagues(30) and the NZB × RF cross,(31) multiple femoral BMD phenotypes were measured. In the B6 × DBA cross by Drake and colleagues, phenotypic data were available for femoral aBMD, femoral volumetric BMD (vBMD), and midshaft vBMD. In the NZB × RF data set, four phenotypes were measured: midshaft vBMD, midshaft cortical vBMD, total femoral vBMD, and total femoral cortical vBMD. In some instances, QTLs with the same peak location and similar CIs were found for more than one femoral phenotype within a single cross. Since there also were unique QTLs for each phenotype, we have presented all QTLs identified for BMD, regardless of type, mapped in these two data sets.

### Comparison with human GWAS loci

To date, 20 substantiated (ie, *p* < 5 × 10^−8^) genome-wide significant (GWS) loci for BMD have been identified in a large meta-analysis of five independent GWAS (the GEFOS Consortium).(3) We compared the locations of these 20 loci with the mouse QTLs (Table 3). We found that 16 of the 20 most significant GWS markers fell within the CI of a mouse QTL. Two of the GWS loci that did not appear, at first examination, to fall within a mouse QTL were very interesting. The first of these, located on human chromosome 3 at 41 Mb, mouse chromosome 9 at 120.7 Mb (72 cM), is only 0.5 cM away from the peak of an acentric QTL mapped in NZB × RF (peak location of 71.49 cM). The second of these, located on human chromosome 6 at 152 Mb, mouse chromosome 10 at 5.7 Mb (2.1 cM), is near the peak of an acentric QTL, 0.4 cM away, mapped in B6 × DBA (peak location of 2.53 cM). In both these cases, the mouse QTL peak location is at or near the last/first marker genotyped in the cross for that chromosome. There are no other informative flanking markers by which to judge if the QTLs are truly acentric. Thus we consider these two human GWS loci likely to be within a mouse QTL. Nine of the human GWS loci are located with 2 cM of the peak location for a mouse QTL (Table 3). An additional four of these loci are within 5 cM of the peak of mouse QTL.

**Table 3:** Comparison of Human GWAS Loci With Mouse QTLs

Human				Mouse				
			
Study (reference)	SNP ID^a^	Chr.	Bp	Chr.	Bp	cM	In mouse QTL?	Nearest mouse QTL (cM)
GEFOS(3)	*rs7524102*	1	22,570,784	4	136,661,533	69.3	Yes	0.2
	*rs6426749*	1	22,583,810	4	136,657,367	69.3	Yes	0.2
	*rs1430742*	1	68,407,413	3	159,552,734	82.7	No	
	*rs2566755*	1	68,407,728	3	159,552,348	82.7	No	
	*rs11898505*	2	54,537,811	11	30,167,162	17.7	Yes	15.3
	*rs87938*	3	41,112,426	9	120,766,377	72.0	Likely	1.0
	*rs1471403*	4	88,994,017	5	104,773,742	50.7	Yes	0.1
	*rs1366594*	5	88,411,567	13	83,453,756	43.7	Yes	1.8
	*rs2941740*	6	152,051,081	10	5,736,893	2.1	Likely	1.0
	*rs2504063*	6	152,132,150	10	5,667,438	2.1	Likely	1.0
	*rs1524058*	7	38,102,552	13	19,538,734	6.9	Yes	0.9
	*rs4729260*	7	95,955,604	6	6,305,527	2.5	Yes	24.9
	*rs7781370*	7	95,971,217	6	6,323,120	2.5	Yes	24.9
	*rs2062377*	8	120,076,351	15	54,166,754	21.1	Yes	0.8
	*rs11995824*	8	120,081,631	15	54,179,867	21.1	Yes	0.8
	*rs7117858*	11	15,650,788	7	122,379,000	60.9	Yes	8.6
	*rs16921914*	11	31,167,097	2	105,937,807	55.5	Yes	2.3
	*rs7932354*	11	46,678,547	2	91,490,042	50.6	Yes	5.9
	*rs599083*	11	67,948,672	19	3,604,166	3.3	Yes	7.7
	*rs2016266*	12	52,013,972	15	102,194,741	57.5	No	
	*rs9533090*	13	41,849,199	14	78,848,972	41.4	Yes	1.3
	*rs10048146*	16	85,267,911	8	123,719,142	70.4	Yes	4.1
	*rs228769*	17	39,548,461	11	102,084,442	66.0	Yes	4.7
	*rs9303521*	17	41,160,727	11	103,944,446	67.7	Yes	3.0
	*rs884205*	18	58,205,587	1	107,744,514	49.7	Yes	1.9
U.S. Caucasians(26,27)	*rs17131547*	1	91,983,358	5	107,591,590	52.1	Yes	1.5
	*rs4276378*	5	7,297,491	13	69251843	35.6	Yes	5.5
	*rs1823926*	5	119,882,620	18	51320749	28.0	Yes	0.4
	*rs11239762*	10	42,582,267	6	118362999	55.9	Yes	2.1
	*rs12437971*	15	98,662,088	7	74,018,517	36.3	Yes	3.1
	*rs16945612*	16	75,986,223	8	116,327,071	60.1	Yes	9.4
KoGES(25)	*rs7776725*	7	120,820,107	6	22,304,412	9.3	Yes	18.1
	*rs550677*	12	124,376,759	5	126,093,513	64.3	Yes	13.1

a Some peaks in the GEFOS Study were denoted by two-peak SNPs. For consistency, both GEFOS peak SNPs are presented. Peaks in the U.S. Caucasians and KoGES studies that were within 1 Mb of peaks in the GEFOS Study are not included.

GWS loci for BMD also have been indentified in smaller cohorts independent of the GEFOS meta-analysis GWAS. While these other cohorts did identify some of the same loci as were found in the GEFOS study, eight additional GWS loci have been described. Deng and colleagues have identified six loci in a small cohort of U.S. Caucasians.(26,27) All six of these loci mapped within the CI of a mouse BMD QTL. Three of these six loci loci mapped to within 2 cM of the peak for a mouse QTL, and a fourth mapped to within 3.1 cM (Table 3). Two more BMD loci were identified in large study of Korean individuals (KoGES Study cohorts).(25) While both these GWS loci were found within the CIs of mouse BMD QTLs, neither of these loci mapped in close proximity to a mouse QTL peak. In the KoGES Study, BMD of the distal radius and of the midshaft of the tibia was measured,(26) not BMD of the femoral neck and lumbar spine, as was measured in the other two GWAS. Three of the KoGES loci were confirmed in the GEFOS Study (human chromosome 7, *rs1721400*, human chromosome 7, *rs6974574*, and human chromosome 13, *rs9525667*) and may represent loci controlling BMD “globally.” The other two KoGES loci may be more site-specific regulators of BMD, and thus it may not be straightforward to compare these loci with the other human loci and with the spinal, femoral, and whole-body BMD QTLs in the mouse.

### Confirmation of *Mef2c* as a candidate gene for BMD

**Fig. 2:**
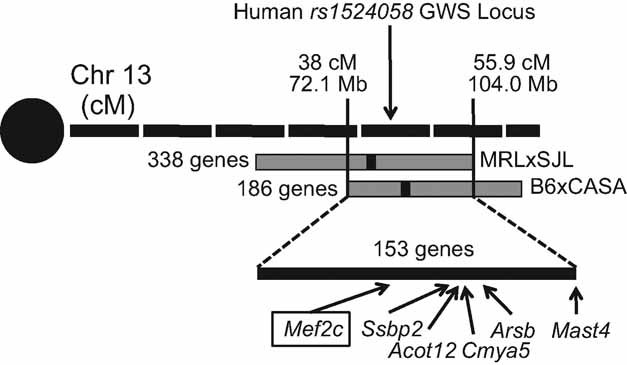
Confirmation of *Mef2c* as candidate gene for BMD on mouse chromosome 13. A GWS locus was identified on human chromosome 7 with a peak at *rs1524058*. The GWS locus is found in close proximity to two BMD QTLs in the mouse that were identified in MRL × SJL and B6 × CASA. The confidence intervals (CI) for these two mouse QTLs overlap for the interval from 38 to 55.9 cM (72.1 to 104 Mb). A total of 338 *Refseq* genes are located within the MRL × SJL CI, 186 genes within the B6 × CASA CI, and 153 genes are located where the CI for these two QTLs overlap. Using block haplotyping, we were able to eliminate all but six of these genes as candidates for this QTL. The gene *Mef2c* (*black box*) is the gene closest to the human GWS locus, and it remained a candidate after haplotyping.

In the GEFOS Study, the most significant SNP at 5q14 for femoral BMD was located in an intergenic region approximately 200 kb distal to the *MEF2C* coding sequence. All the surrounding SNPs with a *p* value of less than 1 × 10^−8^ also were located in this intergenic region, and none of these SNPs is in strong linkage disequilibrium with SNPs in the coding region of *MEF2C.*(3) Furthermore, 5q14 is a novel GWS locus for BMD and has not been replicated thus far in an independent cohort. Thus we wished to substantiate *MEF2C* as a gene that should be further examined for genetic association with BMD. This GWS locus in mice is located between two QTL peaks for aBMD that had been mapped in MRL × SJL and B6 × CASA (Fig. 2). We used block haplotyping to determine if *Mef2c* could be a candidate for these two mouse QTLs. In short, 338 unique *Refseq* genes fall within the confidence interval for the MRL × SJL QTL, 186 in the B6 × CASA QTL, with 153 genes falling in the region wherein the CI for these two QTLs overlap. By block haplotyping, we reduced the list of possible candidate genes to six genes, including *Mef2c* (Fig. 2). We repeated the haplotyping without the SJL/J equal to the CASA/RkJ term and confirmed that *Mef2c* should be considered a candidate for these QTLs in mice.

## Discussion

### Impact of the new genetic map on studies based on congenic mice

A *congenic* is a strain of mice wherein the alleles for specific genetic regions from one inbred strain (the donor strain) are moved over to another inbred strain (the background strain) using a backcross-breeding scheme. Congenic mice are useful in that they can confirm that the alleles of the donor strain at that locus change the phenotype of interest relative to the background strain. Congenics are powerful tools to examine the basic biology underlying a QTL. Congenic mice are genotyped to ensure that the genetic region of interest has been carried over from the donor strain onto the background strain. It is possible to make a congenic region for the “wrong” genetic location (ie, somewhere other than for which the data suggest there is a QTL) and still get a phenotype. QTL analysis is designed to identify association between a genotype and phenotype that achieves some level of statistical significance. Not every locus is going to be identified in a given F_2_ cross, especially if that cross contained only a small number of animals. Congenic studies still in the planning stages could be affected by our finding in that the genetic location of the QTL has “moved.” However, as long as the investigator uses the same markers found to be closest to the QTL peak and the associated flanking markers used in the F_2_ analysis to make the congenic, the correct congenic piece from the donor strain will be carried over.

### Concordant QTLs

One of the main purposes of our study was to provide a listing of BMD QTLs, all mapped using a common genetic map and a common mapping algorithm, such that QTLs from different crosses could be compared with ease and confidence. Furthermore, it has long been thought that there should be concordance in peak location for QTLs identified by different crosses. However, because of discrepancies in the genetic map and in the various calculation algorithms, grouping QTL peaks based on literature-reported peak location was at best tricky and at worse misleading. In this recalculation study, we were not hindered by these obstacles, and we therefore can group QTLs with increased confidence. Indeed, there are clear examples where QTLs identified by various groups may be one and the same. For example, on chromosome 18 we report 10 different QTLs identified in six different crosses (Table 2). Based on peak location, our data suggest that there may be only 4 unique QTLs, not 10. The first of these QTLs is supported only by a single cross, with a peak at 15 cM.(32,33) The second QTL is also supported by only a single cross, the B6 × C3H cross generated by Beamer and colleagues,(34) but the peaks for vertebral vBMD (at 25 cM) and for femoral vBMD (at 29 cM) are likely one QTL regulating global BMD. The peak location for this QTL maps to the same location as a GWAS peak identified by Deng and colleagues.(27) The third QTL group is supported by two different crosses and is represented by a QTL mapped in B6 × 129(35) at 40 cM and by a second QTL mapped in the B6 × C3H cross generated by Farber and colleagues,(29) with a peak at 43 cM. The fourth and final QTL is the most interesting in that it was identified in three crosses: B6 × CAST(36) at 58 cM, B6 × 129(35) at 56 cM, and for three bone phenotypes in NZB × RF(31) at peaks 54, 54, and 56 cM.

A traditional F_2_ cross has poor mapping resolution, so it is difficult to say with confidence how far apart two QTLs can be and still be caused by the same genetic entity. Furthermore, concordant peak location does not mean that the two QTLs share the same genetic cause. There are statistical tests such as biallelic combining of crosses(8) that can be done to interrogate whether two crosses share a QTL or if that QTL is cross-specific. Performing such analysis for all possible shared QTLs is beyond the scope of this study.

### Differences among data sets

While this study does reconcile differences in mapping algorithms and genetic maps, fundamental differences in study design used by different investigators also should be considered. The mice examined in four of the crosses had been fed a high-fat diet prior to obtaining the phenotype data (Table 1). A high-fat diet may alter the BMD, and it is likely that gene-by-dietary-fat interaction(s) may affect BMD as well.(37) Earlier we discuss an example for chromosome 18. The second chromosome 18 QTL grouping is solely represented by B6 × C3H. The third bin also contains a B6 × C3H femoral BMD QTL, but in this case, the mice from the mapping cross had been fed a high-fat diet. In fact, both crosses in this third bin of QTLs had been fed a high-fat diet. Thus the use of a different diet likely suggests that there are indeed two distinct B6 × C3H BMD QTLs on chromosome 18 and that this finding is not a function of poor mapping resolution.

The age of the mice measured for BMD does differ among the 11 data sets analyzed. Peak BMD in mice is obtained at or around 16 weeks of age for most inbred strains.(38) For the majority of data sets, BMD was measured at or near to this age of peak BMD. It must be noted that the mice in the MRL × CAST(39) and MRL × SJL(40) crosses were substantially younger than 16 weeks of age, and for one of the two B6 × DBA crosses, the mice were much older (Table 1). This age difference may explain the differences in QTLs identified in the two B6 × DBA crosses.

For each data set, BMD was obtained using a slightly different protocol. In essence, BMD can be broken down into two types: volumetric BMD (vBMD), as obtained by a computed tomographic approach, and areal BMD (aBMD), for which the BMD was obtained by densitometric analysis of plain-film X-rays or by using a dual-energy X-ray absorptiometer. The BMD data also can be classed by anatomic site of measure: whole-body scans of the head, femur, or vertebra. We list the type of BMD phenotype data for each cross in Table 1. For presentation purposes, we have subdivided the QTLs based on the anatomic site at which BMD was obtained (Fig. 1), but we list the exact BMD phenotype for each QTL in Table 2. Two QTLs with concordant peak locations but for a different anatomic site may well be caused by the same genetic entity, as is suggested earlier by the femoral and vertebral BMD QTLs on chromosome 18 mapped in the B6 × C3H data set from Beamer and colleagues. Other studies, however, have suggested intrinsic differences in bone physiology at different anatomic sites.(41) Thus we have subdivided the QTLs by anatomic site.

### Comparison with human BMD loci

Traditional QTLs for BMD have been mapped in humans in many small studies.(42) Meta-analyses of the human BMD QTLs have been done to improve the power to detect these QTLs.(43,44) In a meta-analysis of genetic loci, the genome is divided into equal-sized bins, and bins with significant linkage to the phenotype are reported. While a valid approach to overcome small-sample-size issues, meta-analysis loci cannot be lifted over easily from one species to another. By definition, the meta-analysis bins are large, and no specific peak location within that bin is calculated. Thus a single-human-genome bin may be homologous to multiple mouse chromosomal regions. For this reason, it is impractical to compare the mouse QTLs with the human BMD meta-analysis results, and we have therefore chosen to compare the mouse QTLs with the human loci mapped in GWAS. The human GWAS loci are sufficiently narrow that they can be lifted over to a single-mouse-chromosomal location, and the GWAS loci reach a genome-wide significant threshold.

There are limitations of the GWAS studies done to date in humans. First of all, no vBMD QTLs have been mapped; only aBMD data are available. Second, sex specificity of the QTLs has not been considered. Third, the X chromosome has not been considered in the GWAS studies for bone. Fourth, GWAS studies consider only the common SNPs (usually minor allele frequency > 1%).(4) Fifth, large GWAS studies for some ethic groups are lacking. Sixth, the highly significant human GWS loci explain only a fraction of the variance found in aBMD in humans.(3) In this study we describe 155 QTLs for mice. As stated earlier, it would be beyond the scope of this article to statistically assess how many of these loci are truly concordant. That said, we estimate that these 155 loci can be collapsed into about 85 distinct loci for BMD identified to date in the mouse. The GWAS studies provide us with a candidate gene(s) to test for only 30% of these QTLs. Finding the genes underlying the remaining 70% of the mouse loci and then testing those genes in humans could be one way of trying to capture some of the “missing variation” or so-called genetic dark matter endemic in GWAS studies.

Work with congenic strains has suggested that there are at least five distinct QTLs on mouse chromosome 1: 37 to 41 cM, 68 to 70 cM, 79 to 80 cM, at 84 cM, and 90 to 97 cM,(45–48) in addition to the B6 × DBA peak at 51.7 cM for femoral aBMD. Only the B6 × DBA peak is likely explained by the GWAS loci identified to date. Edderkaoui and colleagues have determined that Duffy blood group, chemokine receptor (*Darc*), likely explains at least one of these QTL,(49) but this gene has not yet been examined for association with BMD in humans. Lipoxygenase 15 (*Alox15*) was identified as a QTL candidate gene for mouse chromosome 11. This gene was found to be associated with BMD in Chinese women,(50) an ethnic group not yet well represented by a large GWAS. These two examples demonstrate that the mouse can be used to find genes that regulate BMD outside the GWAS loci known to date.

While the resolution of mapping in a GWAS is far superior to the more traditional QTL-based mapping in humans, identification of a GWS locus does not necessary equal immediate identification of a causative gene.(5) Approximately half the human GWS loci for BMD fall in intergenic regions of the genome. Still more GWS loci fall in gene-dense regions, wherein there is more than one candidate gene for the locus.(3) By examining concordant QTLs in mice, we can better resolve these more enigmatic GWS loci to an actual gene or causal variant and make models for the study of the biologic mechanism by which the locus affect BMD. For example, the peak SNP and all the subsignificant supporting SNPs for the human chromosome 5 *rs1366594* GWS locus are located in an intergenic region near to the human *MEF2C* gene.(3) Expression of *Mef2c* has been demonstrated in both osteoblasts and osteocytes in rodents, and *Mef2c* is thought to regulate *Sost* expression, proving a role for this gene in bone biology.(51) Using block haplotyping, we were able to confirm that this gene should be considered a candidate gene for this QTL. Moreover, a potential splice-site polymorphism has been identified in this gene in several strains of mice (*rs47941354*), which may be the underlying causal variant. Body weight and/or body size can influence BMD. Vitarius and colleagues, when originally analyzing the B6 × CASA cross, identified a body-weight QTL on chromosome 13.(52) It is possible that *Mef2c* affects BMD by altering body mass. QTLs for forelimb muscle mass and body length also were examined in the MRL × SJL cross, but no QTLs on chromosome 13 for these two phenotypes are reported.(53) This lack of a lean-mass QTL in the MRL × SJL cross does not rule out a possible role for *Mef2c* in the modulation of fat mass or total body weight to affect bone mass. This possible interaction between body weight and fat mass requires further study. Thus studies in mice can be used to confirm novel GWAS loci, can be used to identify actual causal polymorphisms, and can be used to establish the role of the candidate gene in bone biology.

## Conclusion

In summary, we remapped the QTLs from 11 mouse mapping crosses using a single mapping study design and using a single corrected version of the mouse genetic map. Our results demonstrate that the BMD QTLs found in different crosses bin together in many instances across the genome, validating the use of such QTL narrowing techniques as combined cross-analysis and block haplotyping. We have shown that QTLs mapped with this new version of the mouse genetic map do agree very well with human BMD GWS loci. With the newly available high-resolution GWAS mapping in humans and our improved QTL mapping in mouse, we believe that this is an opportune time for a renewed effort by the genetics community to identify the causal variants regulating BMD using a synergistic mouse-human approach.
